# Phenotypes and outcome of diffuse pulmonary non-amyloid light chain deposition disease

**DOI:** 10.1186/s12931-024-02798-y

**Published:** 2024-04-10

**Authors:** François Lestelle, Catherine Beigelman, David Rotzinger, Salim Si-Mohamed, Mouhamad Nasser, Lidwine Wemeau, Sandrine Hirschi, Grégoire Prevot, Antoine Roux, Vincent Bunel, Emmanuel Gomez, Laurent Sohier, Helene Morisse Pradier, Martine Reynaud Gaubert, Anne Gondouin, Romain Lazor, Jean-Charles Glerant, Françoise Thivolet Bejui, Magali Colombat, Vincent Cottin, the OrphaLung network, Yurdagül Uzunhan, Stéphane Jouneau

**Affiliations:** 1 Hospices Civils de Lyon, Centre de Référence Coordinateur Des Maladies Pulmonaires Rares (OrphaLung), Hôpital Louis Pradel, Service de Pneumologie, 69677 Lyon, France; 2 Service de Radiologie Et de Radiologie Interventionnelle, Hôpital Universitaire de Lausanne, Université de Lausanne, Lausanne, Suisse; 3 Hospices Civils de Lyon, Hôpital Louis Pradel, Service de Radiologie, Lyon 69677U1206, Université de Lyon, INSA-Lyon, Université Claude Bernard Lyon 1, UJM-Saint Etienne, CNRS, Inserm, CREATIS, UMR 5220, F‐69621, 7 Avenue Jean Capelle O, 69100, Villeurbanne, France; 4 Centre de Référence Constitutif Des Maladies Pulmonaires Rares (OrphaLung), CHU Lille, Service de Pneumologie, Lille, France; 5 Centre de Compétence Des Maladies Pulmonaires Rares (OrphaLung), CHU Strasbourg, Service de Pneumologie, Strasbourg, France; 6 Centre de Compétence Des Maladies Pulmonaires Rares (OrphaLung), CHU Toulouse, Hôpital LarreyUniversité Paul Sabatier, Toulouse, France; 7 Service de Pneumologie Et de Transplantation Pulmonaire, Hopital Foch, Suresnes, France; 8 Service de Pneumologie B Et de Transplantation Pulmonaire, AP-HP, Hôpital Bichat Claude-Bernard, Inserm U1152, Paris, France; 9 Centre de Compétence Des Maladies Pulmonaires Rares (OrphaLung), CHU Nancy, Service de Pneumologie, Nancy, France; 10 Centre Hospitalier Bretagne Sud, Service de Pneumologie, Lorient, France; 11 Centre de Compétence Des Maladies Pulmonaires Rares (OrphaLung), CHU Rouen, Service de Pneumologie, Rouen, France; 12 Service de Pneumologie Et Transplantation Pulmonaire, CHU Marseille Nord, Aix-Marseille Université Marseille, Assistance Publique-Hôpitaux de Marseille, Marseille, France; 13 Centre de Compétence Des Maladies Pulmonaires Rares (OrphaLung), CHU Besançon, Service de Pneumologie, Besançon, France; 14 Service de Pneumologie, Centre Hospitalier Universitaire Vaudois, Lausanne, CH Suisse; 15 Hospices Civils de Lyon, Hôpital Louis Pradel, Service d’explorations Fonctionnelles Respiratoires, 69677 Lyon, France; 16 Hospices Civils de Lyon, Hôpital Louis Pradel, Service d’Anatomopathologie, 69677 Lyon, France; 17 CHU Toulouse, Institut Universitaire du Cancer de Toulouse, Service d’anatomie Et Cytologie Pathologiques, Toulouse, France; 18 UMR754, INRAE; Member of RespiFil and ERN-LUNG, Université, Claude Bernard Lyon 1, Lyon, France

**Keywords:** Light chain deposition disease, Lung cysts, Bronchiectasis, Lung transplantation

## Abstract

**Background:**

Light chain deposition disease (LCDD) is a very rare entity. Clinical manifestations of LCDD vary according to the organs involved. Data on pulmonary LCDD are scarce and limited to small series or case reports. This study aimed to describe the characteristics and outcome of diffuse pulmonary non-amyloid LCDD localized to the lungs.

**Study design and methods:**

A multicenter retrospective cohort study was conducted. Clinical characteristics were collected, and chest CTs were centrally reviewed. The diagnosis of pulmonary non-amyloid LCDD was confirmed by immunohistochemistry.

**Results:**

Thirty-one cases were identified (68% female), with a median age at diagnosis of 50 years (IQR 20). Baseline FEV1/FVC was < 0.70 in 45% of patients. Mean (**± **SD) FEV1 and DLCO were 86% ± 26.2 and 52% ± 23.9, respectively. CT revealed peculiar patterns of thin-walled cysts (58%) and thin-walled cystic bronchiectases (27%). Increased serum kappa light chain was found in 87% of patients. Histological analysis showed kappa light chain deposits in all patients, except one with lambda chain deposits. Median annual FEV1 decline was 127 ml (IQR 178) and median DLCO decline was 4.3% (IQR 4.3). Sixteen patients received immunomodulatory treatment or chemotherapy; serum light chain levels decreased in 9 cases (75%), without significant improvement in FEV1 (*p* = 0.173). Overall, 48% of patients underwent bilateral lung transplantation. Transplant-free survival at 5 and 10 years were 70% and 30%, respectively. An annual FEV1 decline greater than 127 ml/year was associated with increased risk of death or transplantation (*p* = 0.005).

**Conclusions:**

Diffuse pulmonary LCDD is characterised by female predominance, a peculiar imaging pattern with bronchiectasis and/or cysts, progressive airway obstruction and severe DLCO impairment, and poor outcome. Lung transplantation is a treatment of choice.

**Supplementary Information:**

The online version contains supplementary material available at 10.1186/s12931-024-02798-y.

## Plain language summary

Diffuse pulmonary light chain deposition disease is an exceedingly rare disease of the lungs, whereby parts of abnormal antibodies (proteins involved in host defense especially against microorganisms) deposit in the lungs. This disease is characterised by a peculiar pattern on chest CT, with dilation of bronchi and formation of air-filled cysts (holes), and progresses in several years to chronic respiratory insufficiency.

## Take-home message

Diffuse pulmonary light chain deposition disease is characterised by female predominance, a peculiar imaging pattern with bronchiectasis and/or cysts, progressive airway obstruction and diffusion capacity impairment, and poor outcome.

## Background

Immunoglobulins can cause specific forms of lung involvement. Their physiochemical properties and size are important pathogenetic determinants. Two forms of immunoglobulin light chains (LC) can be deposited in tissues: amyloid [1] and non-amyloid. Light chain deposition disease (LCDD) is a term restricted to the non-amyloid forms of LC deposition.

LCDD is a rare multisystemic entity described by Randall in 1976 [2] as the deposition of a nonfibrillary, amorphous material that does not have a β-pleated sheet configuration and consequently does not bind Congo red nor have apple-green birefringence with Congo red stain. Contrary to LC amyloidosis [3], LCDD is mostly composed of kappa LC. Moreover, electronic microscopy does not show a fibrillary pattern but electron-dense granular deposits along basement membranes [4]. The diagnosis of LCDD is established by immunohistological analysis of affected organs. It requires a formalin-fixed paraffin-embedded sample for microscopic examination and a frozen sample for immunofluorescence analysis with anti-kappa and anti-lambda antibodies. When a frozen tissue sample is not available, mass spectrometry on formalin-fixed paraffin-embedded tissue can be used [5].

Clinical manifestations of LCDD vary according to the organs involved. Lung involvement appears to be very uncommon but may be underrecognised especially when the deposition of LCs is limited to the lung. Since its first report in 1988 [6], pulmonary LCDD has been described as either nodular or diffuse [7, 8]. The nodular form is generally seen in patients who have no evidence of plasma cell dyscrasia. Diffuse LCDD is characterised by parenchymal cysts, or airway involvement [9, 10] including bronchiectasis [11]. Pathologically, diffuse LCDD is characterised by LC deposits along the basement membranes of the alveolar, bronchial, and vascular walls. The putative pathophysiology of cyst formation involves the degradation of elastic fibers by matrix metalloproteinases [12].

The clinical symptoms reported in previous studies are chest discomfort, haemoptysis, and progressive dyspnea leading to chronic respiratory failure [13–15]. Although no treatment is validated, chemotherapy is often prescribed to control monoclonal LC secretion in the serum [16]. Lung transplantation may be performed [17].

Data on pulmonary LCDD are scarce and limited to small series or case reports. The main objectives of this study were to: 1) describe the clinical, functional and radiological characteristics at presentation, 2) determine lung function during follow-up and estimate time to transplantation or death.

## Methods

### Patient selection and data collection

This retrospective multicentre study was conducted in the French OrphaLung network, a cooperative group of lung specialists. Patient data regarding clinical, laboratory, functional, radiological characteristics, and outcome were collected using a case report form. Patients were considered eligible if the diagnosis of pulmonary LCDD was confirmed by immunohistochemistry. The exclusion criteria were uncertain diagnosis, solitary pulmonary nodules, and predominance of amyloid deposits.

### Ethical consideration

This study was conducted with respect to the Declaration of Helsinki. It was approved by the ethics committee of the *Hospices Civils de Lyon* and was registered with the national data protection agency (Commission Nationale de l’Informatique et des Libertés, number 20–075). According to the legislation in place at the time of the study, informed consent signature was waived, but each patient was informed by a written letter and could object to the use of their personal data. Several patients were reported in previous publications [10–12, 14, 17, 18].

### Pulmonary function tests

Lung volumes were measured by plethysmography, and forced vital capacity (FVC) and forced expiratory volume in one second (FEV1) by flow–volume curve, using GLI reference equations. Carbon monoxide transfer factor (DLco) was assessed with the single-breath method. Hypoxemia was defined as a partial pressure of oxygen in arterial blood (PaO2) < 80 mmHg.

### Chest computed tomography (CT)

Two expert chest radiologists (SSM, DR) blinded to the clinical data reviewed the baseline and latest available computed tomography (CT) images during follow-up and before lung transplantation. If consensus was not obtained, a third expert radiologist (CB) settled the description. A CT grading system was used to document cyst number, size of the largest cyst, shape (round, oval, irregular), internal septation, and distribution. Bronchiectases were classified by their appearance (cylindric, varicose, cystic), their proximal or distal distribution, and the presence of bronchial wall thickening. Then, based on imaging, the predominant imaging pattern was classified as bronchiectatic, cystic, or mixed. To evaluate temporal changes in imaging findings, the last available follow-up CT was compared to the baseline CT.

### Pathological assessment

Histology reports of lung biopsies were collected and centrally reviewed by an expert pathologist in the field (MC) to confirm the diagnosis of LCDD, using previously described criteria [5], i.e. biopsies had to stain negative with Congo red and demonstrate LC deposits on frozen tissue under immunofluorescence microscopy. When frozen tissue was unavailable, mass spectrometry was used for diagnostic confirmation [19].

### Statistical analysis

Although normal distribution could not be assumed due to the small sample size and the expected heterogeneity in disease behavior, Shapiro–Wilk test was used to check for normal and skewed distributions. Non-parametric tests were used in the absence of normality. Continuous variables are presented as means (percentages) ± standard deviation (SD) or as median (range).

The Wilcoxon signed-rank test was applied to compare pre- and post-treatment lung function decline, and the Mann–Whitney U test was used to compare measures between groups. Chi-squared test, Fisher’s exact test and Student’s t test were used where appropriate. For each patient, the estimated annual decline rate in DLCO and FEV1 expressed in %/year and in mL/year, respectively, were calculated by linear regression. The average ∆FEV1 and ΔDLCO were calculated in patients who had completed a minimum of 24 months of follow-up and a minimum of four measures. Event-free survival, defined as the time from the first consultation to transplantation or death from any cause, was estimated using the Kaplan–Meier survival method, and univariate Cox regression was performed to assess hazard ratios (HRs) with 95% confidence intervals.

Statistical significance was set at *p* < 0.05 (two-tailed). IBM SPSS Statistics for Windows, Version 25.0. (IBM Corp, Armonk, NY) and RStudio (v1.3.959) were used for statistical analyses.

## Results

### Baseline characteristics

Out of 61 patients identified between 1998 and 2020 in 12 French centres and one Swiss centre, 31 met the eligibility criteria and were included in the analysis (Figure S1). Overall, 68% of patients were women and the mean ± SD age at diagnosis was 50 ± 10.7 years. The median (range) interval between the onset of symptoms and diagnosis was 4 (1–30) years. Twenty (67%) patients were current or former smokers with a median of 23.8 (2.5–120) pack-years. All patients except two had dyspnea (Table 1).

**Table 1:** Patient characteristics at baseline

	**Value**	**Patients with available data, n**
Demographic data		
Age at symptom onset, years, median (IQR)	42.5 (17.5)	30
Age at diagnosis, years, median (IQR)	50 (20)	29
Female sex, %	68	31
Smoking history, %	67	30
Pack-years, median (IQR)	15 (17.5)	19
Clinical data, n (%) Dyspnea, %	91	30
Cough, %	80	30
Sputum, %	70	30
Haemoptysis, %	30	30
History of recurrent bronchitis or pneumonia, %	64.5	30
History of extrapulmonary infectious disease, %	6.7	30
History of spontaneous pneumothorax (≥ 1), %	17	30
Palpable lymphadenopathy, %	10	30
Laboratory data		
IgG, mg/L, mean ± SD (normal reference)	9.8 ± 4.57 (7.00—16.00)	22
IgA, mg/L, mean ± SD (normal reference)	1.9 ± 0.82 (0.78- 4.11)	21
IgM, mg/L, mean ± SD (normal reference)	3.66 ± 3.78 (0.40- 2.80)	22
Increased serum kappa/lambda ratio, %	91	23
Mean serum kappa/lamba ratio ± SD	30.3 (**± **43.8)	22
Increased kappa light chain in serum, %	87	23
Mean kappa light chain in serum, mg/L ± SD	236.2 (**± **344.4)	23
Increased lambda light chain in serum, %	0	22
Mean lambda light chain in serum, mg/L ± SD	11.4 (**± **5.8)	21
Alpha 1 anti-trypsin deficiency, %	0	11
Lung function parameters at baseline		
FEV1, L, mean ± SD	2.5 ± 0.78	29
FEV1, % pred, mean ± SD	86.2 ± 26.7	27
FVC, L, mean ± SD	3.3 ± 1	28
FVC, % pred, mean ± SD	92.3 ± 23	27
FEV1/FVC, %, mean ± SD	74.9 ± 11.8	27
TLC, L, mean ± SD	5.93 ± 1.78	26
TLC, %, pred, mean ± SD	105.9 ± 16.8	23
DLCO, % pred, mean ± SD	52.5 ± 23.9	22
KCO, % pred, mean ± SD	57 ± 27	18
PaO2, mmHg, mean ± SD	73.3 ± 13.4	19
6 min walk test, m, mean ± SD	503 ± 103	12

*FEV1* forced expiratory volume in one second, *FVC* forced vital capacity, *TLC* total lung capacity, *DLCO* carbon monoxide transfer factor, *IQR* interquartile range, *KCO* transfer coefficient for the lung for carbon monoxide, *PaO2* arterial oxygen partial pressure, *%pred*, percentage of predicted value

At baseline, FEV1/FVC was < 0.70 in 45% of patients. FEV1 was lower than 80% of the predicted value in 43% of patients. DLCO was 52 ± 24% (Table 1). Hypoxemia was present in 68% of patients.

### Haematological characteristics and biology

Bone marrow biopsy or aspiration performed in 21 cases showed > 10% of plasma cells in three patients. A bone marrow B-cell clone search was performed in seven cases and was found in four. Immunofixation identified a circulating monoclonal component as IgM in 52% of cases. Kappa/lambda ratio of serum LC was increased in 21 patients. Free monoclonal kappa LC was increased in the serum of 21/23 patients with a mean level of 236.2 (**± **344.4) mg/L (Table 1). An auto immune workup, including antinuclear and antineutrophilic cytoplasmic antibodies, performed in 22 patients, was negative in all but one patient with anti-SSA and anti-SSB antibodies.

### Histology and immunohistochemistry

The most frequently used method to obtain tissue was videothoracoscopic lung biopsy (36%) (Figure S2A). The pathological examination of lung biopsies demonstrated cystic destruction and bronchiolar dilatation in all cases (Figure S2B). In all patients, biopsy specimens showed Congo red negative eosinophilic deposits infiltrating alveolar walls, small airways, and/or vessels. Immunofluorescence assay of frozen tissues was performed in 25 cases, showing LC deposition stained using anti-kappa antibodies in 19 patients, as illustrated in Figure S2C. In contrast, anti-lambda antibody staining confirmed the diagnosis in only one patient. Electron microscopy, performed in five patients, revealed granular dense electron deposits in all cases. Mass spectrometry-based analysis of biopsy, performed in 19 cases, showed that the main constituent of the deposits in all cases was the presence of peptides belonging to the constant region of the immunoglobulin kappa chain. A lymphoplasmocytic infiltrate was found in 13 cases. A search for B-cell clone in the lung was performed by PCR in 9 cases and revealed a clone in all patients.

### Fiberoptic bronchoscopy

Fiberoptic bronchoscopy was performed in 24 patients. The macroscopic appearance of the bronchial mucosa was inflammatory in 29% of patients, normal in 42%, showed bronchial distorsions in 12.5%, and bronchomalacia in 8%. Bronchial biopsies were performed in 11 cases, including 8 with immunohistochemical analysis (positive in 5), and 3 with proteomic analysis (positive in all 3 cases).

### Baseline CT characteristics

The most frequent abnormalities were plurifocal and sometimes extensive lung cysts (100%) and cystic bronchiectasis (77%). Cysts were bilateral and of regular shape and were characterised by internal septation (73%), bronchovascular topography (77%), and thin walls (Supplement Figure S3). There was a wide variation in cyst size; the largest cysts were found abutting the pleura (Table 2). The patient population was then split according to whether patients presented with a cystic, bronchiectasis, or mixed CT pattern. FEV1 was significantly lower, and airflow obstruction was significantly more severe in the cystic pattern compared to the bronchiectasis pattern (Table 3).

**Table 2:** Chest computed tomography findings in 26 patients

**Feature**	n (%)
**Cysts**	26 (100%)
Shape	
Oval	2 (8%)
Irregular	3 (11%)
Round	6 (23%)
No predominance	15 (58%)
Internal septation	19 (73%)
Peribronchovascular	20 (77%)
Vertical distribution	
Upper predominance	3 (11.5%)
Middle predominance	3 (11.5%)
Lower predominance	14(54%)
No predominance	6 (23%
Axial distribution	
No predominance	17 (70%)
Central	6 (23%)
Peripheral	3 (12%)
Number	
< 50	7 (27%)
50–100	6 (23%)
> 100	13 (50%)
Location of largest cysts	
Abutting the pleura	21 (80%)
Surrounded by lung parenchyma	5 (20%)
**Bronchiectasis**	20 (77%)
Shape	
Cystic	1 (5%)
Cylindric	0
Varicose	19 (95%)
Vertical Distribution	
No predominance	10 (56%)
Upper predominance	0
Lower predominance	8 (44%)
Horizontal distribution	
Proximal predominance	1 (5%)
Distal predominance	19 (95%)
Bronchial wall	
Normal	3 (12%)
Thin	17 (65%)
Thick	6 (23%)
**Bronchocele, mucus plug**	9 (37%)
**Predominant imaging pattern**	
Cystic	15 (58%)
Bronchiectasis	7 (27%)
Mixed	4 (15%)
**Nodules (< 1 cm in diameter)**	11 (42%)
**Lymphadenopathy**	
Supracentimetric mediastinal lymph node	4 (15%)
Infracentimetric mediastinal lymph node	16 (61%)
Hilar lymph node	9 (37%)
**Consolidation**	5 (19%)
**Emphysema**	9 (35%)

**Table 3:** Comparison of patient characteristics according to bronchiectasis or cystic pattern predominance

	**Bronchiectasis pattern (*n* = 7)**	**Cystic pattern (*n* = 15)**	**p**
Demographic data			
Age at onset of symptoms, mean (**± **SD)	50 ± 6	44 ± 13	0.54
Age at diagnosis, mean (**± **SD)	56 ± 6	48 ± 12	0.16
Female sex, n (%)	6 (85)	6 (40)	0.07
Smoking history, n (%)	4 (57)	12 (80)	0.33
Pneumothorax history, n (%)	0	3 (20)	0.5
Lung function parameters at baseline			
FEV1, % pred, mean (**± **SD)	107 ± 17	86 ± 26	0.04
FVC, % pred, mean (**± **SD)	103 ± 19	93 ± 24	0.33
FEV1/FVC, %, mean (**± **SD)	83 ± 4.7	74 ± 10	0.01
Airflow obstruction, n (%)	0	7 (46)	0.05
TLC, %pred, mean (**± **SD)	98 ± 11	108 ± 17	0.17
DLCO, % pred, mean (**± **SD)	62.2 ± 19.8	54 ± 24	0.33
KCO, %pred, mean (**± **SD)	54.5 ± 33.4	67 ± 20	0.36
6 min walk test, m, mean (**± **SD)	565 ± 86.7	496 ± 92	0.16
Change in lung function parameters			
Mean ∆FEV1, ml/year (**± **SD)	184 ± 139	216 ± 227	0.96
Mean ∆DLCO, %/year (**± **SD)	2.76 ± 1.4	4.59 ± 2.7	0.27
Laboratory data			
Mean kappa light chain in serum, mg/L (**± **SD)	192 ± 205	326 ± 441	0.75
Mean lambda light chain serum, mg/L (**± **SD)	9 ± 4.6	11 ± 6.4	0.53

*FEV1* forced expiratory volume in one second, *FVC* forced vital capacity, *TLC* total lung capacity, *DLCO* carbon monoxide transfer factor, *KCO* transfer coefficient for the lung for carbon monoxide, *PaO2* arterial oxygen partial pressure, *%pred* percentage of predicted value

### Extrapulmonary manifestations

In four cases, LC deposits were found in salivary glands, one case being associated with primary Sjögren syndrome. Kidney tissue was available in three cases and no deposit was found. Interventricular septum thickening on cardiac ultrasonography was found in none of the cases. No patient had congestive heart failure. Left ventricular ejection fraction was preserved in all cases, and diastolic dysfunction was absent. Low voltage was not present on electrocardiograms. Right heart catheterisation was performed in 11 patients (Supplement Table S1).

### Lung function and CT follow-up

**Fig. 1:**
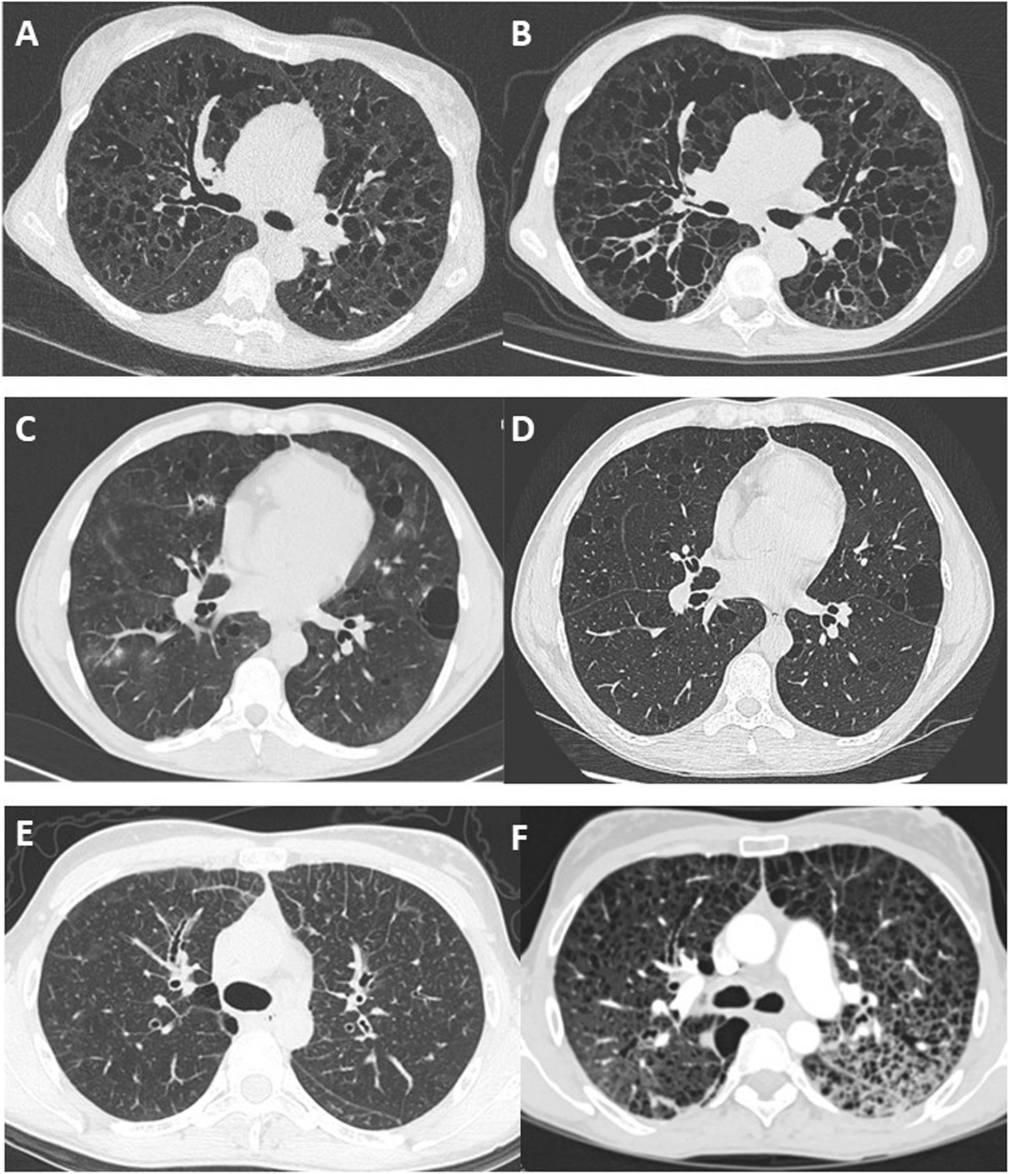
Long-term change in chest CT features in 3 patients showing 3 patterns. **A** and **B** CT scan at baseline and after 8 years in a patient with diffuse cystic bronchiectasis. **C** and **D** CT scan at baseline and after 1 year in a patient with regular round cysts of various sizes. **E** and **F** CT scan at baseline in a patient with micronodules and interlobular reticulation, and 7 years later with multiple cysts and ground glass attenuation

Follow-up data were available for FEV1 in 26 patients and for DLCO in 17 patients. The median duration of lung function follow-up was 75 (2–180) months. The median annual overall FEV1 decline was 127 ml/year (2.2 – 877), and the median DLCO annual decline was 4.3%/year (1.9 – 8). Imaging follow-up was available for 16 patients with a median interval between the first and last chest CT of 62 (7–139) months. Cysts increased in size in 13 patients and in number in ten patients (Fig. 1). The number of bronchiectases increased in nine patients.

### Treatment

Nonpecific treatment left to the discretion of each investigator included inhaled glucocorticoids (*n* = 16), long-acting beta-agonists (*n* = 15), long-acting muscarinic antagonist (*n* = 6), long-term macrolide treatment (*n* = 9). In 16 cases, a treatment was prescribed to attempt treating the disease by targeting the underlying production of LC. In all cases except 4, the first line of chemotherapy was different for each patient, varying according to centres (Supplementary figure S4).

Among the 16 patients treated, ten had evaluable measurements of serum-free LC before and after systemic treatment. A normalisation or reduction > 90% of serum-free LC kappa was obtained only in three patients. Only eight patients had evaluable annual FEV1 before and after systemic treatment. The annual decline in FEV1 did not differ significantly between the pre-and post-treatment periods (*p* = 0.73; Supplementary Table S2).

Long-term supplemental nasal oxygen was used in 19 patients (66%). Fifteen patients underwent double lung transplantation, with a median interval between diagnosis and lung transplantation of 5 years (1–11). In 4/15 cases, the diagnosis of LCDD was made before the transplantation. No patient requiring a lung transplant was excluded from receiving it due to contraindications and no patient died on the lung transplant waiting list.

### Survival and prognostic factors

When comparing patients deceased or transplanted with those alive or not transplanted, there were significant differences in age at onset of symptoms, baseline FEV1% predicted, FEV1/FVC, and ∆FEV1 (Table 4).

**Table 4:** Univariate analyses of the predictive factors of outcome (death or lung transplantation)

	**Hazard Ratio**	**95% CI**	***p***
Demographic data			
Age at onset of symptoms, mean	0.945	0.904—0.988	0.013
Age at diagnosis, mean	0.942	0.902—0.983	0.0057
Sex	0.714	0.435—1.169	0.18
Smoking status	0.955	0.352—2.587	0.93
Lung function parameters			
FEV1, % pred	0.974	0.955—0.994	0.012
FVC, % pred	0.985	0.962 -1.01	0.21
FEV1/FVC, %	0.942	0.903—0.982	0.005
TLC, % pred	1.023	0.989—0.982	0.19
DLCO, % pred	0.971	0.942—1.002	0.06
KCO, % pred	0.980	0.945—1.015	0.26
6-min walk test, m	0.995	0.987 – 1.003	0.21
Mean ∆FEV1, ml/year	1.007	1.003 – 1.011	0.00095
Mean ∆DLCO, %/year	1.039	0.827 – 1.305	0.75
Serum light chains			
Mean kappa light chain in serum, mg/L	1.001	0.999 – 1.002	0.30
Mean lambda light chain in serum, mg/L	1.016	0.898 – 1.150	0.80
Mean serum kappa/lambda ratio	1.008	0.997 – 1.019	0.14
Predominant pattern (cystic/bronchiectasis)	0.491	0.226 – 1.069	0.07

*FEV1* forced expiratory volume in one second, *FVC* forced vital capacity, *TLC* total lung capacity, *DLCO* carbon monoxide transfer factor, *PaO2* arterial oxygen partial pressure, *%pred* percentage of predicted value

**Fig. 2:**
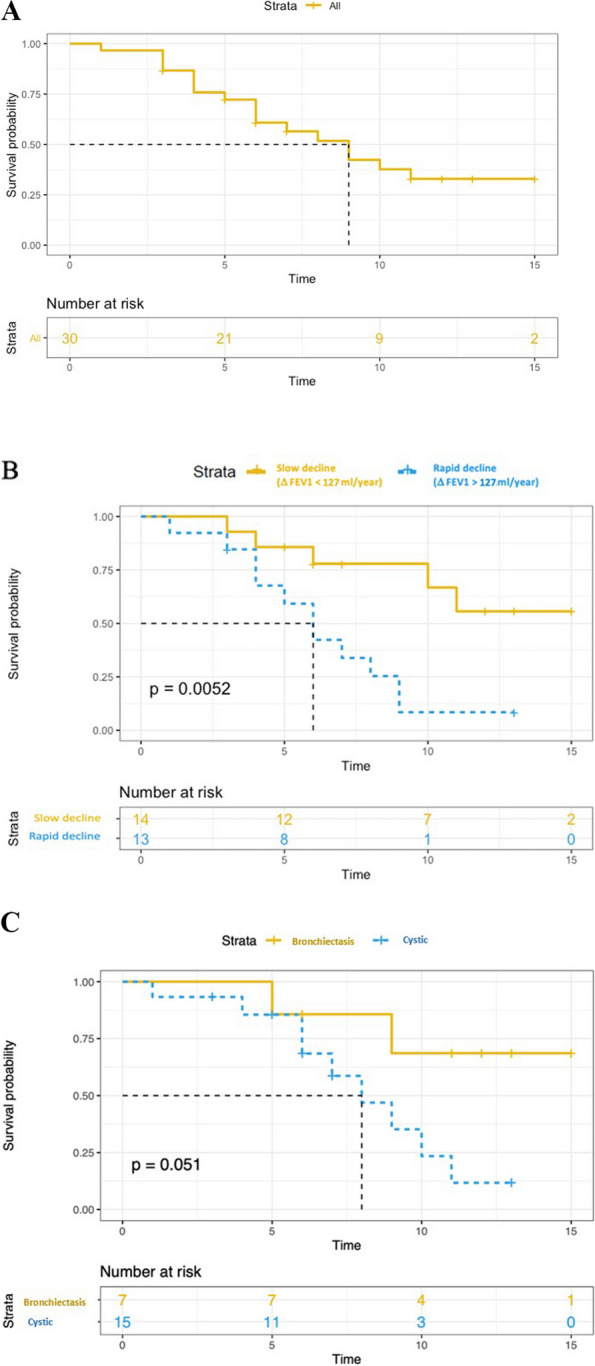
Kaplan–Meier estimates of transplant-free survival. **A** transplant-free survival in all patients. **B** Yellow and blue curves correspond to Kaplan Meier estimates of the transplant-free survival for patients with FEV1 decline greater or less than 127 ml/year (*P* value for log rank test). **C** Yellow and blue curves correspond to Kaplan Meier estimates of the transplant-free survival for patients with “bronchiectasis pattern” and “cystic pattern”, respectively (*P* value for log rank test)

Three patients died at the age of 45, 55, and 68 years, all of them due to progressive respiratory failure. The median transplant-free survival was 9 years (Fig. 2). There was no significant difference in transplant-free survival between men and women, between smokers and non-smokers, between treated and non-treated, or between cystic and bronchiectatic patterns. A rapid decline in FEV1, defined as ∆FEV1 > 127 ml/year (median value), was associated with an increased risk of death or transplantation (*p* = 0.005) (Fig. 2).

## Discussion

The present cohort of diffuse pulmonary LCDD, which is the largest to date, shows a female predominance, an increase in serum free monoclonal kappa LC at diagnosis, and a predominance of thin-walled lung cysts and bronchectases on CT. Moreover, pulmonary LCDD appears as a progressive airflow obstruction associated with a poor prognosis and limited response to chemotherapy. Double lung transplantation should be considered as a curative option in this population, and patients should be referred early for transplantation.

Diffuse pulmonary LCDD localized to the lungs is less well described than other cystic lung diseases such as lymphangioleiomyomatosis or pulmonary Langerhans cell histiocytosis. In the patients’ files, the disease was often reported as “unclassifiable diffuse cystic lung disease”, “idiopathic diffuse bronchiectasis”, or “atypical emphysema” before LCDD was considered. The diagnosis is probably often missed since IF is rarely applied to lung biopsies by pulmonary pathologists and frozen material is rarely available, although it is considered the diagnostic method of choice. In these situations, mass spectrometry may confirm the diagnosis, but is not widely available. Paraffin IF was not performed in the present cohort but a prior study suggested that this technique is feasible and could rescue some difficult diagnoses [20]. The pathological diagnosis was based on the findings of videothoracoscopic lung biopsy, bronchial biopsy, and/or lung explant. Interestingly, bronchial biopsy tissue analysis was sufficient to confirm the diagnosis in a large proportion of the patients in whom the diagnosis was suspected, and in the cases where IF could be performed on frozen bronchial biopsies. Hence, physicians should be alerted to use adequate tissue fixation methods (i.e. frozen tissue) when a diagnosis of LCDD is considered.

Previous studies clearly demonstrated that diffuse pulmonary LCDD differs clinically from the systemic multivisceral form and from the pulmonary nodular form of the disease [21]. Unlike patients in two recent reports [22, 23], the patients herein rarely had underlying autoimmune conditions, in particular primary Sjögren syndrome. Nodular pulmonary LCDD may be associated with Sjögren syndrome more often than the cystic presentation [24].

The present results indicate that young females more frequently present with respiratory manifestations of LCDD. The most common lung function abnormality at baseline was decreased DLCO, probably related to the damage induced by LC deposits along the alveolar-capillary membrane, as observed in lung amyloidosis [25]. Airflow obstruction defined by FEV1/FVC < 70% was also common.

The CT findings of the present study were relatively heterogeneous. In contrast to the findings of Sheard et al. [26], pulmonary involvement primarily consisted of varicose bronchiectasis without nodular infiltrates, except in one case. The coexistence (and possible communication [12]) of thin-walled bronchiectasis and cystic changes is somewhat characteristic of diffuse pulmonary LCDD when compared to other cystic lung diseases, in particular lymphangioleiomyomatosis and Birt-Hogg-Dubé syndrome. Cysts in pulmonary LCDD are regular in shape with a predominance of lower distribution [27]. During follow-up, the size and the number of bronchiectases and pulmonary cysts increased remarkably. However, in some cases, distinguishing these two patterns was not possible, with extensive emphysema-like changes in severe forms, and it is conceivable that both bronchiectases and cyst result from a common mechanisme of elastolysis. Noteworthy, the majority of the patients herein were current or ex-smokers, and it cannot be excluded that tobacco smoking may have played a role in pulmonary lesion formation.

Monoclonal gammopathy is the presence in the serum of a monoclonal immunoglobulin produced by a B-cell clone. This biological anomaly fits with the concept of monoclonal gammopathy of clinical significance, in which the clone induces severe organ damage [28]. Herein, the free LC ratio was abnormal in the vast majority of patients, making it a plausible screening tool. Nevertheless, the serum level of kappa LC at baseline was not associated with a greater probability of lung transplant or death. As shown by the plasmacytosis greater than 10% found in certain patients who underwent bone marrow biopsy or aspiration, although the association of LCDD with haematological malignancy is possible [13, 29], the absence of bone marrow abnormality upon immunohistology should not exclude pulmonary LCDD. In such cases, LC may be produced within the lungs [17]. No evidence of extrapulmonary LC deposition was observed in the present cohort, except in accessory salivary glands. As a result, pulmonary LCDD may be considered in the appropriate context, even in the absence of kidney dysfunction or proteinuria.

During follow-up, inter-patient variability in FEV1 decline was observed, however, the decline in lung function was generally faster than that seen in chronic obstructive pulmonary disease [30] or in other multiple cystic lung diseases [31–33], and diffusion capacity was severely altered. Patients who required a lung transplantation had a higher rate of FEV1 decline than non-transplanted patients. Consequently, lung function surveillance at 6–12 months intervals is of paramount importance to assess disease progression and identify possible transplantation candidates, especially when ΔFEV1 > 127 ml/year.

There is no validated therapeutic strategy for LCDD localized to the lung. The main treatment approach is to target the synthesis of monoclonal proteins, using the same treatments as those employed in multiple myeloma. In the present cohort, various medications were used (rituximab, alkylating agents, proteasome inhibitor, dexamethasone, antimetabolite) targeting the underlying production of light chains, as well as autologous blood stem cell transplantation. Using an exploratory analysis, no change in the annual rate of FEV1 decline before and after treatment was observed, whereas improved renal function was reported in patients treated with melphalan and prednisone [34] or autologous stem cell transplantation for renal LCDD [35]. The fact that tissue damage induced by LC deposits is irreversible may explain the absence of clinical improvement despite serum LC titers that tended to decrease upon treatment. It remains to be determined whether treatments allowing LC serum levels to return to normal may slow disease progression. Lung volume reduction surgery might be considered as an alternative to chemotherapy in selected cases [18].

Lung transplantation is currently the main treatment option in patients with respiratory failure resulting from LCDD, with no reported recurrence in the transplanted lungs [17]. This is in line with the relatively long median lung transplant-free survival found herein. However, the present analysis showed that there was a significant difference in lung-transplant free survival according to FEV1 decline, underlying the possible need for some patients to undergo transplantation as early as possible. In contrast, kidney transplantation is not considered an option in end-stage renal disease, as further kidney damage cannot be prevented in the transplanted organ [36]. In case of cardiac LCDD, LC deposits were identified as early as 3 months after heart transplantation [37].

This study has several limitations, including the relatively small sample size and the retrospective design. Second, its multicentric nature represents a source of variability in pulmonary function test measurements. Third, the lack of standardised therapy may have contributed to the lack of significant treatment benefit observed on lung function decline.

## Conclusions

In conclusion, in this cohort of patients with diffuse pulmonary LCDD, women were more commonly affected than men. Low DLCO was the most commonly observed lung function abnormality. Rapid annual FEV1 decline was associated with a greater risk of death or transplantation. Systemic haematologic treatment did not reduce the annual lung function decline. Prospective larger studies are eagerly awaited.

### Supplementary Information

**Supplementary Material 1.**

## Data Availability

Data are available upon request to the corresponding author.

## Abbreviations

CT: Computed tomography
DLCO: Diffusing capacity for carbon monoxide
KCO: *Transfer coefficient for the lung for carbon monoxide*
FVC: Forced vital capacity
FEV1: Forced expiratory volume in one second
IF: Immunofluorescence
LC: Light chain
LCDD: Light chain deposition disease
PaO2: Arterial oxygen partial pressure
TLC: Total lung capacity
