# Supplementary material for: Phenotypes and outcome of diffuse pulmonary non-amyloid light chain deposition disease
Source: Respir Res. 2024 Apr 10;25:159. doi: 10.1186/s12931-024-02798-y (PMC11005206; doi:10.1186/s12931-024-02798-y)
Supplement: Supplementary file 1 — Supplementary Material 1. [file 12931_2024_2798_MOESM1_ESM.zip › Supplementary/2024_02_10 NAMIDD_Respir_Res_Supplement_R1_clean.docx]

**Phenotypes and outcome of diffuse pulmonary non-amyloid light chain deposition disease**

*Supplementary material*

François Lestelle ^1^, Catherine Beigelman ^2^, David Rotzinger ^2^, Salim Si-Mohamed ^3^, Mouhamad Nasser ^1^, Lidwine Wemeau ^4^, Sandrine Hirschi ^5^, Grégoire Prevot ^6^, Antoine Roux ^7^, Vincent Bunel ^8^, Emmanuel Gomez ^9^, Laurent Sohier ^10^, Helene Morisse Pradier ^11^, Martine Reynaud Gaubert ^12^, Anne Gondouin ^13^, Romain Lazor ^14^, Jean-Charles Glerant ^15^, Françoise Thivolet Bejui ^16^, Magali Colombat ^17^, Vincent Cottin ^1,18^ and the OrphaLung network

**Results**

Right heart catheterisation was performed in 11 patients and found a mean pulmonary artery pressure (mPAP) >20 mmHg in 10 patients. Pulmonary wedge pressure (PAWP) was available in 6 cases and was <15 mmHg in all of them (Supplement Table S1). Precapillary pulmonary hypertension (defined by mean mPAP > 20 mmHg with pulmonary artery wedge pressure ≤ 15 mmHg and pulmonary vascular resistance > 3 Woods Units) was observed in 4 patients. Acute vasoreactivity was tested in one case and was positive. This patient was treated by calcium channel inhibitor.

**Supplementary Table S1.** Haemodynamic characteristics. Data are presented as mean SD.

|  | **Value** | **Cases with available data** |
| --- | --- | --- |
| **Echocardiography** |  |  |
| Estimated sPAP > 35 mmHg, % | 81 | 16 |
| **Right heart catheterization** |  |  |
| Mean mPAP, mmHg | 26.2 ± 6.0 | 11 |
| Mean PAWP, mmHg | 9.3 ± 3.4 | 6 |
| Mean PVR, WU | 2.8 ± 1.1 | 6 |
| Mean CI, L/min/m2 | 3.4 ± 0.7 | 9 |

*sPAP: systolic pulmonary artery pressure; mPAP: mean pulmonary artery pressure ; PAWP: pulmonary artery wedge pressure; PVR: pulmonary vascular resistance; CI : cardiac index.*

**Supplementary Table S2:** Lung function and laboratory characteristics before and after systemic treatment in 16 patients.

|  | **Before** | **After** | **p** |
| --- | --- | --- | --- |
| Lung function parameters |  |  |  |
| FEV1, % pred, mean | 94 ± 21 | 82 ± 26 | 0.053 |
| FVC, % pred, mean | 102 ± 20 | 95 ±20 | 0.08 |
| FEV1/FVC, % pred, mean | 76 ± 9 | 70 ± 15 | 0.048 |
| TLC, %pred, mean | 107 ± 14 | 113 ± 14 | 0.06 |
| DLCO, % pred, mean | 55 ± 17 | 38 ± 19 | 0.002 |
| KCO, % pred, mean | 54 ± 15 | 44 ± 15 | 0.005 |
| mean ∆FEV1, ml/year (n=8) | 127 ± 105 | 145 ± 123 | 0.73 |
|  |  |  |  |
| Serum light chains |  |  |  |
| Mean kappa light chain in serum, mg/L | 311 ± 314 | 120 ± 155 | 0.13 |
| Mean lambda light chain serum, mg/L | 12 ± 6 | 14 ± 16 | 0.68 |
|  |  |  |  |

*FEV1: forced expiratory volume in one second; FVC: forced vital capacity; TLC: total lung capacity; DLCO carbon monoxide transfer factor; KCO: transfer coefficient for the lung for carbon monoxide; %pred percentage of predicted value.*

**Legend of supplementary Figures**

**Supplementary Figure S1.** Flowchart of the study.

**Supplementary Figure S2**. Pathological assessment. (A) Frequency of lung tissue sampling methods expressed in percentage; (B) cystic destruction of the lung in an explant specimen; (C) kappa light chain staining along alveolar walls using immunofluorescence (white arrows). Lambda staining was negative.

**Supplementary Figure S3.** (A) An axial CT image focused on the left upper lobe showing a regular cyst with regular shape (black arrow) and varicose bronchiectasis with very thin wall (white arrow). (B) and (C) Axial and coronal chest CT images showing multiple thin-walled cysts (black arrow) and bronchiectasis (white arrows) distributed on both sides. Interlobular septal thickening may be prominent.

**Supplementary Figure S4**. Systemic treatment regimens in 16 patients who received first-line exclusive chemotherapy, possibly followed by second-line, third line, and fourth-line systemic treatment, from the most external to the most inner ring. Four patients received an anti-CD20 monoclonal antibody, together with an alkylating agent. Ten patients received two or more consecutive treatment lines. DXM: dexamethasone
