# Supplementary figures and images for: Phenotypes and outcome of diffuse pulmonary non-amyloid light chain deposition disease
Source: Respir Res. 2024 Apr 10;25:159. doi: 10.1186/s12931-024-02798-y (PMC11005206; doi:10.1186/s12931-024-02798-y)

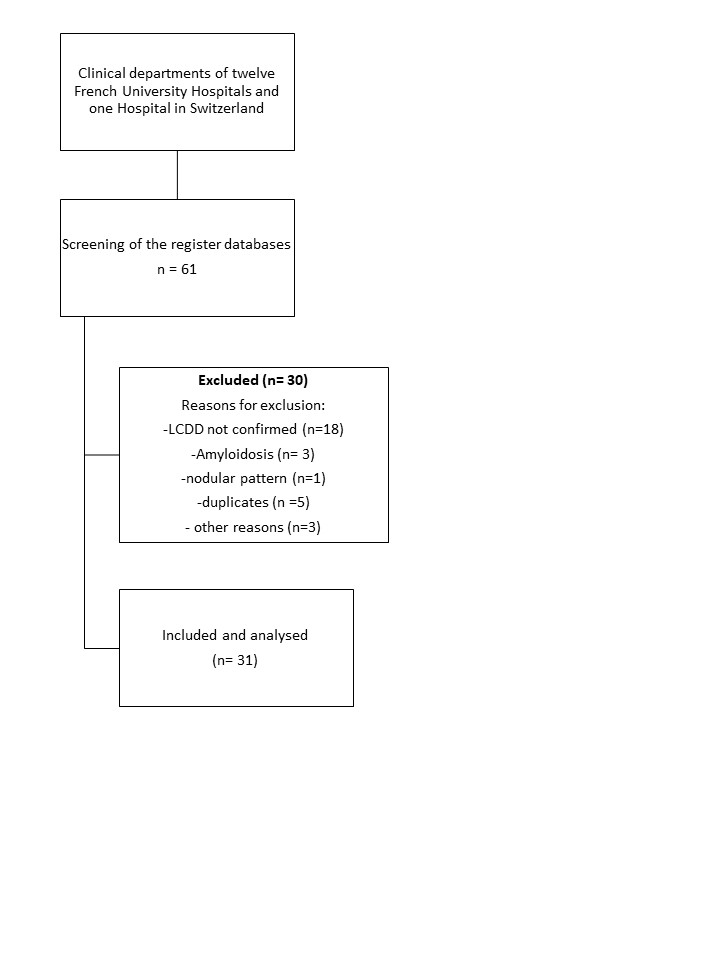

Supplement: Supplementary file 1 — Supplementary Material 1. [file 12931_2024_2798_MOESM1_ESM.zip › Supplementary/Figure S1 Flowchart.jpg]

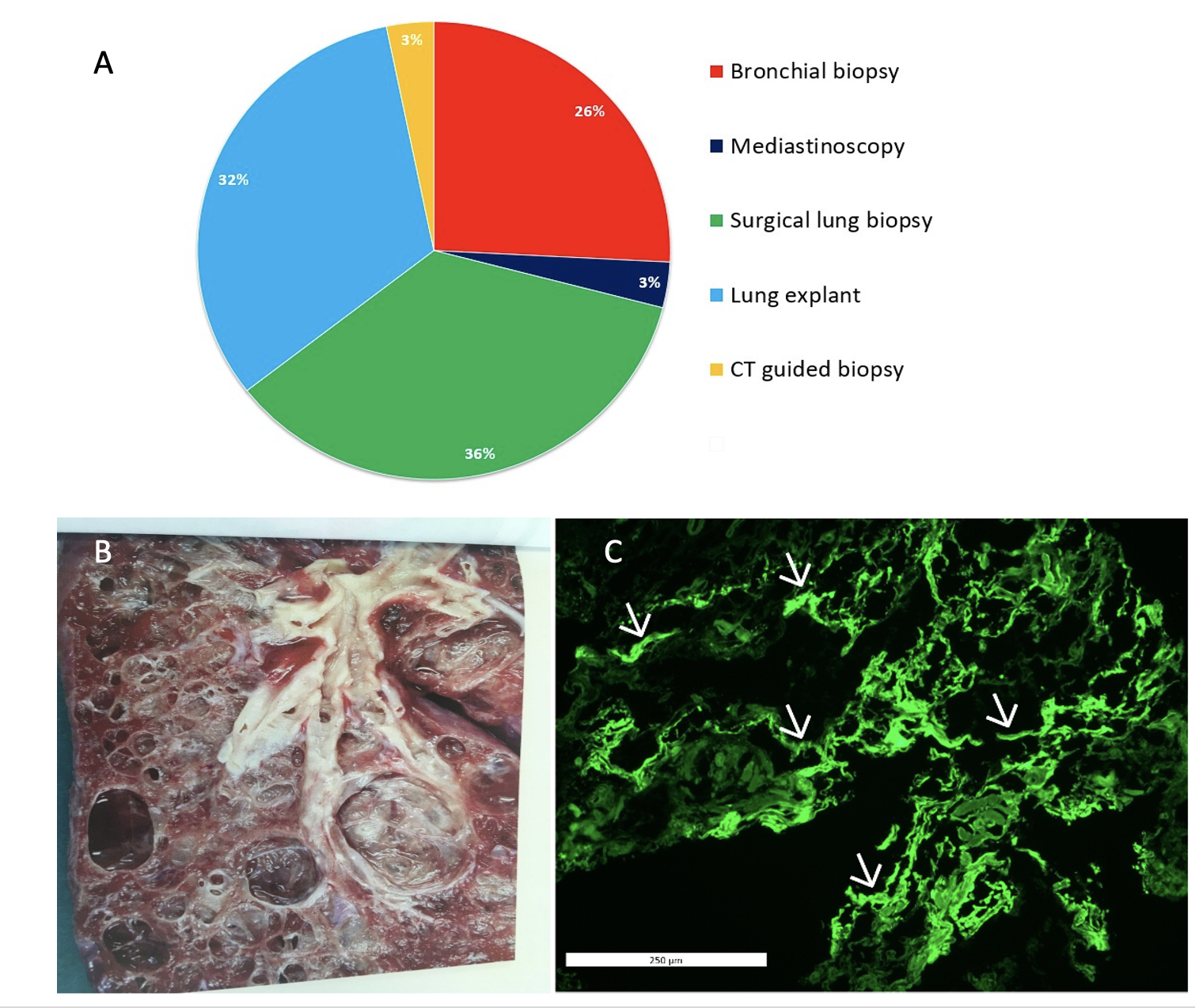

Supplement: Supplementary file 1 — Supplementary Material 1. [file 12931_2024_2798_MOESM1_ESM.zip › Supplementary/Figure S2 pathology revised.png]

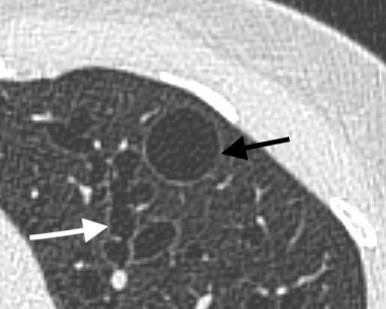

Supplement: Supplementary file 1 — Supplementary Material 1. [file 12931_2024_2798_MOESM1_ESM.zip › Supplementary/Figure S3A CT revised.jpg]

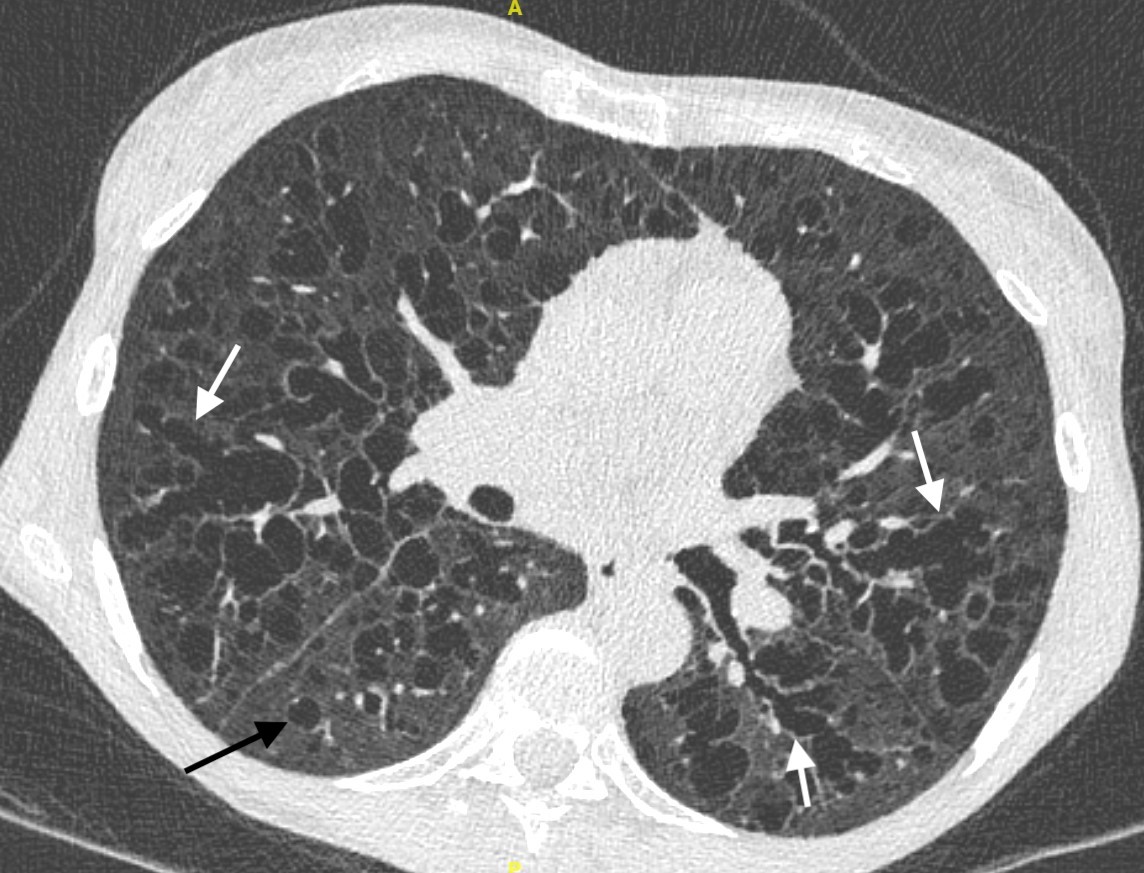

Supplement: Supplementary file 1 — Supplementary Material 1. [file 12931_2024_2798_MOESM1_ESM.zip › Supplementary/Figure S3B CT revised.jpg]

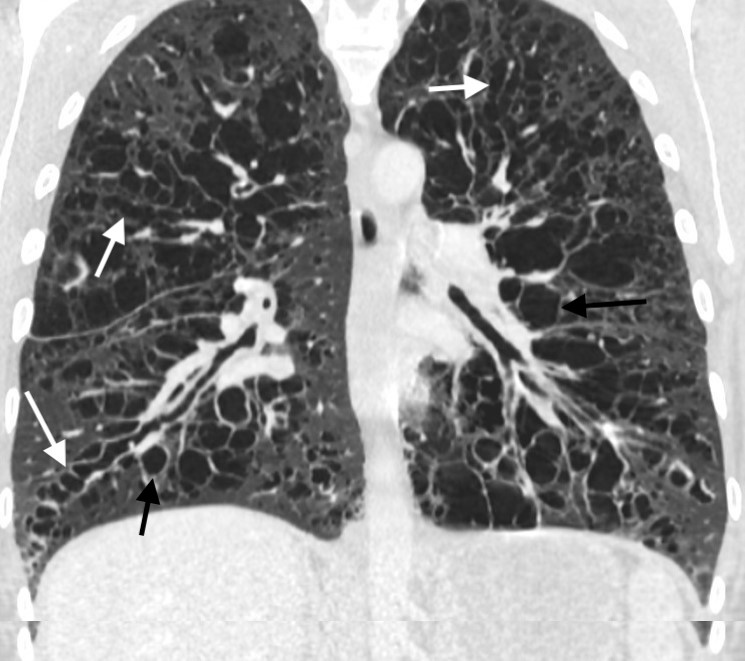

Supplement: Supplementary file 1 — Supplementary Material 1. [file 12931_2024_2798_MOESM1_ESM.zip › Supplementary/Figure S3C CT revised.jpg]

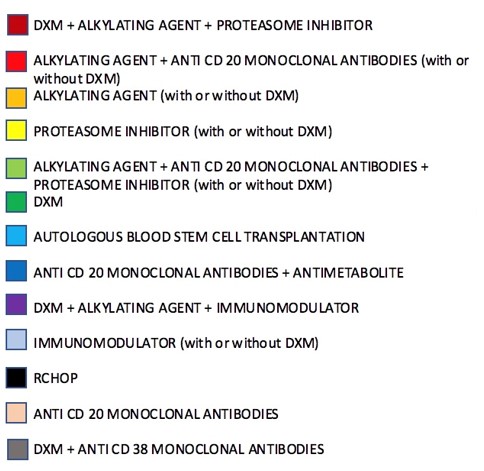

Supplement: Supplementary file 1 — Supplementary Material 1. [file 12931_2024_2798_MOESM1_ESM.zip › Supplementary/Figure S4 TT Legend.jpg]

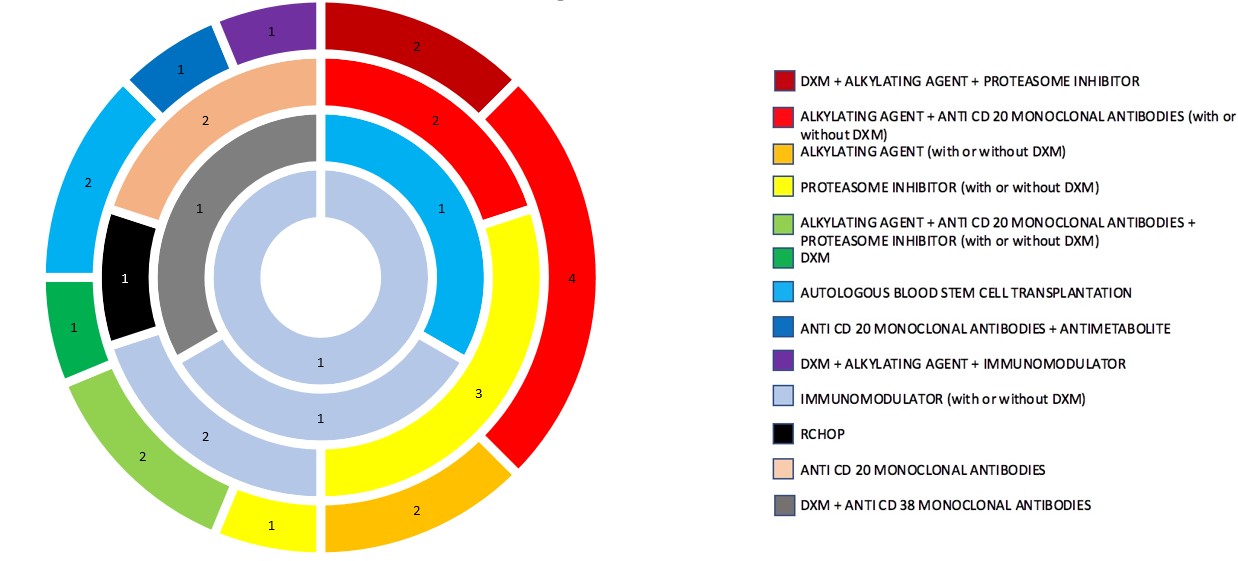

Supplement: Supplementary file 1 — Supplementary Material 1. [file 12931_2024_2798_MOESM1_ESM.zip › Supplementary/Figure S4 TT.jpg]
